# Reverse causal reasoning: applying qualitative causal knowledge to the interpretation of high-throughput data

**DOI:** 10.1186/1471-2105-14-340

**Published:** 2013-11-23

**Authors:** Natalie L Catlett, Anthony J Bargnesi, Stephen Ungerer, Toby Seagaran, William Ladd, Keith O Elliston, Dexter Pratt

**Affiliations:** 1 Selventa, One Alewife Center, Cambridge, MA 02140, USA

## Abstract

**Background:**

Gene expression profiling and other genome-scale measurement technologies provide comprehensive information about molecular changes resulting from a chemical or genetic perturbation, or disease state. A critical challenge is the development of methods to interpret these large-scale data sets to identify specific biological mechanisms that can provide experimentally verifiable hypotheses and lead to the understanding of disease and drug action.

**Results:**

We present a detailed description of Reverse Causal Reasoning (RCR), a reverse engineering methodology to infer mechanistic hypotheses from molecular profiling data. This methodology requires prior knowledge in the form of small networks that causally link a key upstream controller node representing a biological mechanism to downstream measurable quantities. These small directed networks are generated from a knowledge base of literature-curated qualitative biological cause-and-effect relationships expressed as a network. The small mechanism networks are evaluated as hypotheses to explain observed differential measurements. We provide a simple implementation of this methodology, Whistle, specifically geared towards the analysis of gene expression data and using prior knowledge expressed in Biological Expression Language (BEL). We present the Whistle analyses for three transcriptomic data sets using a publically available knowledge base. The mechanisms inferred by Whistle are consistent with the expected biology for each data set.

**Conclusions:**

Reverse Causal Reasoning yields mechanistic insights to the interpretation of gene expression profiling data that are distinct from and complementary to the results of analyses using ontology or pathway gene sets. This reverse engineering algorithm provides an evidence-driven approach to the development of models of disease, drug action, and drug toxicity.

## Background

Molecular profiling technologies have enabled the collection of large, exploratory data sets consisting of measurements for tens of thousands of molecular entities. These rich data sets hold promise for understanding the molecular bases of disease, drug action, and drug toxicity, but do not often lead to a reasonable short list of potential molecular mechanisms that can be investigated further by targeted experiments. For example, gene expression profiling experiments frequently result in a list of hundreds or thousands of gene expression differences that characterize a comparison of biological states like diseased versus normal tissue or treatment versus control. The development of methods to interpret these lists of differential measurements and extract testable hypotheses is necessary to realize the full potential of these large data sets.

The use of prior knowledge in the form of functional groupings of genes into gene sets is central to many methods for interpreting molecular profiling data. Generally, a collection of gene sets is assessed to identify those for which differentially expressed genes from the data set are over-represented (reviewed in [1-3]). Genes can be grouped into sets based on a variety of criteria including: (1) functional annotation, (2) pathway maps, (3) regulatory or structural motifs, and (4) common response to an experimental perturbation.

Analyses using gene sets based on pathway maps (e.g., KEGG, [4]) or functional annotation (e.g., Gene Ontology, [5]) rely on the assumption that differential RNA expression is equivalent to differential protein activity. This assumption can be problematic because RNA abundances are not generally a good measure for the abundance or activity of the corresponding proteins. The correlation of RNA expression to protein expression is variable, with changes in RNA levels accounting for only a modest portion of changes in levels of the corresponding proteins [6-8]. As a group, signal transduction proteins are more poorly correlated with their corresponding RNAs than are structural proteins, thus extrapolation of RNA microarray data to infer changes in signal transduction requires caution [6,7]. Protein abundance is regulated via translation and proteolysis in addition to regulation via transcription and stability of mRNA. Moreover, protein expression levels are frequently not the key determinant of protein activity; many pathways are regulated by post-translational events such as protein modification or binding, and only secondarily by protein abundance.

Experimental result-based gene sets, for example the L2L, GeneSigDB, and MsigDB chemical and genetic perturbations (CGP) gene sets [9-11], do not evaluate differentially expressed RNAs based on the function of the proteins they encode, but rather by a common mechanism controlling their expression (e.g., the experimental perturbation). This type of gene set has the additional advantage that the function of the corresponding gene products need not be well defined to be considered. One limitation of experimental result-based gene sets is that the sets of genes up-regulated and down-regulated within an experiment are often either handled as distinct “up” and “down” sets or combined without regards to direction. Additionally, gene sets from experiments with similar chemical or genetic perturbations are not generally integrated into a single cohesive gene set.

In this paper, we present a detailed description of Reverse Causal Reasoning (RCR), a reverse engineering algorithm to identify biological mechanisms that are statistically significant explanations for differential measurements in a molecular profiling data set. This approach uses prior knowledge in the form of a large network of biological cause and effect relationships, from which smaller networks representative of discrete biological mechanisms are derived. These smaller networks are essentially structured gene sets, where the upstream node represents an experimental perturbation like a chemical, protein, or protein activity, the downstream nodes represent entities such as RNAs that have been measured in the data set, and the edges specify an “increased”, “decreased”, or “ambiguous” relationship. These mechanism networks are evaluated for both overrepresentation and consistency with respect to differential measurements from a data set.

This general approach has been in use by Selventa since 2005 to identify and evaluate molecular mechanisms involved in diverse biological processes, but not formally described or tested using a publically available knowledge base [12-19]. A related approach has been recently reported [20]. In addition, an approach using the same network structure to represent mechanisms has been developed to quantify and allow comparison of the level of activity of a mechanism between related data sets [21].

We provide an implementation of RCR, Whistle (available at https://github.com/Selventa/whistle), specifically designed for analysing gene expression profiling data, and apply Whistle to three example gene expression profiling data sets. Whistle uses prior knowledge in the form of Biological Expression Language (BEL) statements compiled into a causal graph. BEL is a language for representing causal and correlative biological relationships from the scientific literature in a computable form. The BEL Framework, which provides tools for compiling and managing BEL statements, has recently been made available to the general public as open source along with a starter corpus of statements, used in this paper to analyse the example data sets (https://github.com/OpenBEL/openbel-framework).

## Methods

### Overview of RCR and whistle

RCR interprets molecular profiling data sets by inferring a set of molecular mechanisms with associated direction (increased or decreased) that can serve as potential causes for the observed profiling differences between two sample groups (e.g., RNAs differentially expressed in diseased vs. normal tissue). This algorithm requires a large knowledge base consisting of prior biological knowledge in the form of cause-and-effect relationships, where the entities measured by the molecular profiling experiment are included as targets of the cause-and-effect relationships.

**Figure 1:**
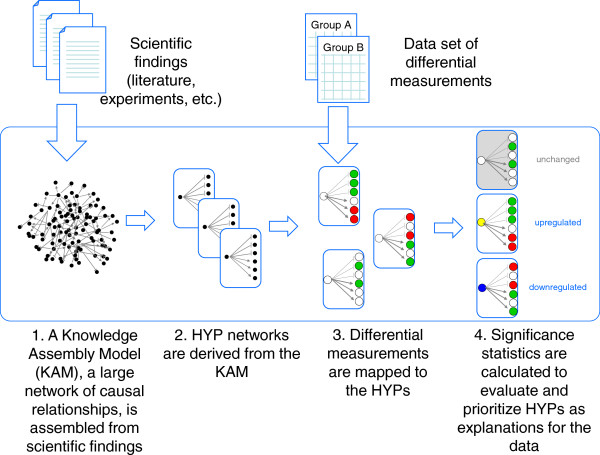
**Overview of whistle.** Whistle evaluates molecular mechanisms as potential explanations for gene expression data by mapping measurements and differentially expressed genes to a directed network of prior scientific knowledge.

Whistle is an implementation of RCR that uses a BEL knowledge assembly model (KAM) as the knowledge base to analyse a gene expression profiling data set comparing two sample groups. Whistle uses the following general strategy (Figure 1):

1. A KAM is assembled from biological findings

2. Small networks representative of a mechanism (HYPs) are derived from the KAM; each KAM node immediately upstream of a minimum number of measured RNA abundance nodes represents a HYP.

3. Differential mRNA abundance measurements are mapped to mechanism networks and a direction (increased, decreased, or none) is assigned for each network.

4. Evaluation statistics (*richness* and *concordance*) are calculated for each mechanism network.

Term definitions related to Whistle input and output are provided in Table 1.

**Table 1:** Whistle term definitions

**Term**	**Definition**
HYP	A small, directed causal network comprised of a single upstream node representing a biological entity or process connected by a causal increase, decrease, or ambiguous edges to downstream nodes representing measured entities.
KAM	Knowledge Assembly Model. A knowledge base of biological cause-and-effect relationships in the form of a network.
Population	The set of measured RNA abundances present in the KAM and included in the *possible* for at least one HYP.
State changes	RNA abundances in the *population* assigned a state of significant increase or decrease, based on the data set.
Direction	Inferred state of the upstream node of a HYP, based on the states of the downstream nodes. Possible values are increased, decreased, and none.
Possible	The number of downstream nodes for a given HYP, representing measured RNA abundances.
Correct	The number of significantly increased or decreased downstream nodes for a given HYP that are consistent with the inferred *direction*.
Contra	The number of significantly increased or decreased downstream nodes for a given HYP that are inconsistent with the inferred *direction*.
Ambiguous	The number of significantly increased or decreased downstream nodes for a given HYP that are connected to the upstream node by an ambiguous edge.
Observed	The total number of significantly increased or decreased downstream nodes for a given HYP.
Richness	A HYP evaluation statistic characterizing the enrichment of significantly increased or decreased downstream nodes in a HYP relative to the *population*. Calculated using the hypergeometric distribution.
Concordance	A HYP evaluation statistic characterizing the consistency of significantly increased or decreased downstream nodes.

### Whistle evaluates prior knowledge as HYPs

**Figure 2:**
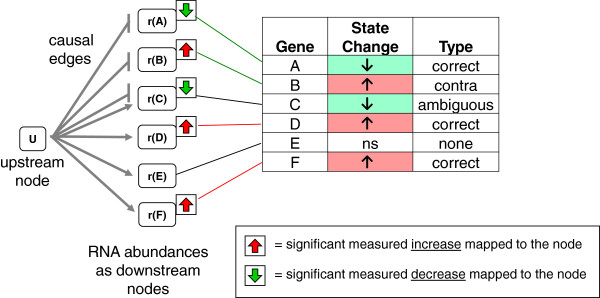
**Mapping of differential measurements to a HYP network.** A HYP consists of an upstream node, U, and downstream nodes, designated r(A) – r(F), that represent abundances of RNAs measured in the experiment. This example network has six measured downstream nodes (*possible*), five of which map to significantly increased or decreased genes (*observed*). Node E is not significantly changed in expression. Three nodes, r(A), r(D), and r(F), support increased U (*correct*). One node, r(B), supports decreased U (*contra*). One node, r(C), is connected to U by both causal increase and causal decrease edges (*ambiguous*). On the basis of the mapped measurements, the direction “increased” is assigned to U.

The prior knowledge structures used by Whistle are termed ‘HYPs’ (derived from hypotheses). A HYP is a small, directed acyclic network containing an upstream node and downstream nodes that map to measured entities (Figure 2). A HYP can be compared to a qualitative Bayesian network or qualitative probabilistic network [22,23]. Each edge in a HYP represents a qualitative influence of increases (+), decreases (−), or ambiguous (?). Unlike qualitative probabilistic networks, zero influences (0) are not included within HYPs. Like a naïve Bayes classifier, all downstream nodes in a HYP are assumed independent from each other, given a fixed value of the upstream node; the downstream nodes are assumed to be uncorrelated with each other.

In the case of Whistle, HYPs are essentially gene sets structured as directed networks, such that both the enrichment of differentially expressed genes and consistency of the directions (increased, decreased) can be evaluated. Each Whistle HYP is derived from a KAM. An *increases* edge connecting two nodes within a HYP means that the nodes are connected by an *increases* or *directlyIncreases* BEL relationship in the KAM. Similarly, a *decreases* HYP edge is derived from a *decreases* or *directlyDecreases* KAM relationship. An *ambiguous* HYP edge means that the KAM contains multiple, conflicting edges connecting an upstream and downstream node, e.g., both an *increases* and *decreases* edge. Other classes of HYPs, e.g., those using other edge types, paths consisting of multiple edges, and/or other types of measured downstream nodes, can be derived from KAMs and evaluated by RCR, but they are not described here or evaluated by Whistle.

### Mapping probe sets to the KAM

First, Whistle maps the set of measured RNAs for a data set to the KAM. This mapping is used to generate HYPs for evaluation, determine the downstream nodes for each HYP (*possible*), and determine the set of downstream nodes for all HYPs evaluated (the *population)*.

Probe set names or other identifiers (e.g., gene symbol or Entrez gene ID) are mapped to RNA abundance nodes in the KAM using the equivalence files provided by the BEL Framework (see http://wiki.openbel.org/ for documentation of BEL Framework namespaces and equivalence files). Next, each KAM node with at least four causally downstream measured RNA abundance nodes is selected as the upstream node for a HYP, resulting in a set of HYPs from the KAM for evaluation against the data set.

### Mapping differential measurements to HYP networks

Next, the data set of differential measurements is mapped to the HYP networks and basic metrics including *direction*, *correct*, and *contra* are calculated (see Table 1). Differential gene expression data is processed such that each measurement is expressed as one of three discrete states: significant increase, significant decrease, or no significant change, based on user-defined significance criteria. These criteria generally include thresholds for fold change, adjusted p-value, and average abundance (see *Data analysis for examples*).

A *direction* of increased, decreased, or none is assigned to each HYP, representing the inferred state of the upstream node of the network based on the states of the downstream nodes mapped from the data set. The *direction* is the state consistent with the majority of the significantly increased and decreased downstream nodes of the HYP network. For each significantly increased or decreased HYP downstream node, the causal linkage(s) to the upstream node determines if the observed state is consistent with an increased, decreased, or neither (ambiguous) state of the upstream node. For example, if upstream node A is connected to RNA abundance node B by a causal decrease, a significant increase in B would be consistent with a decrease in A. If upstream node A is connected to B by an ambiguous edge, an increase in B would be considered an ambiguity.

### Evaluation statistics (concordance and richness)

Two evaluation statistics, *concordance* and *richness*, are calculated for each HYP. These statistics allow the HYPs to be prioritized based on the strength and consistency of their support in the data set. Lower concordance and richness p-values reduce the number of false positive, but increase the number of false negative HYP inferences. As is common for other prior knowledge-driven approaches [3], both the concordance and richness statistics are biased in favour of HYP networks with a larger number of downstream nodes. For most statistic-based metrics, values associated with a larger number of measurements can achieve higher levels of significance than those associated with a small number of measurements (see *Results -Bias in evaluation statistics by HYP size*). Thus, we use these metrics primarily as a filter to identify potential explanations for the gene expression data, and not as a strict ranking for the most interesting explanations. A threshold of p < 0.1 for both *concordance* and *richness* generally limits both false negatives and false positives to an acceptable level, and provides a manageable set of HYPs for manual evaluation (see *Results*).

### Concordance

The *concordance* statistic is calculated as a p-value that characterizes the consistency of the observed states of the downstream nodes with the direction assigned to the HYP upstream node. Concordance is calculated as a binomial distribution where the directions expected for the downstream RNA abundance nodes, based on the assigned direction of the HYP upstream node and the causal relationships to the downstream nodes are tested. Downstream nodes with the assigned state no significant change and nodes connected to the upstream node by an ambiguous relationship are excluded. For the set of significantly increased or decreased nodes comprising the *correct* and *contra* for a HYP, the *concordance* statistic is the probability of getting at least the number of state changes consistent with the direction (*correct*).

The binomial distribution can be written as *f*(*k;n,p*) where:

*k* is the number of successful predictions (*correct*),

*n* is the number of trials (*observed*), and

*p* is the probability of achieving a result, 0 ≤ p ≤ 1.

The point probability of getting exactly *k* successful predictions is calculated as:

$$\mathrm{prob}=f\left(k;n,p\right)=\left(\begin{array}{c} n\\ k \end{array}\right)p^{k}{\left(1-p\right)}^{n-k}$$

For the purposes of evaluating a HYP, the probability of getting the direction of prediction correct, *p*, is 0.5; the network can either predict the observed direction for a state change correctly or incorrectly. For some HYP *x*_*i*_, the number of trials, *n*_*i*_, corresponds to the number of downstream nodes for the mechanism that are mapped to state changes (*observed*). The number of successful predictions *k*_*i*_ is the number of downstream nodes of *x*_*i*_, mapped to state changes that are consistent with the HYP direction (*correct*). Let *l*_*i*_ be the number of downstream nodes for which the predicted direction cannot be determined (*ambiguous*).

The point probability for some HYP, *x*_*i*_, for some *k*_*i*_ is then calculated as:

$$\mathrm{pro}b_{\mathrm{ik}}=\left(\begin{array}{c} n_{i}-l_{i}\\ k_{i} \end{array}\right)p^{k_{i}}{\left(1-p\right)}^{n_{i}-k_{i}-l_{i}}$$

The *concordance* p-value is a cumulative probability based on the area under the curve of a probability distribution function. Thus, concordance for some HYP, *x*_*i*_, is the sum of *prob*_*ij*_ for all *j* = *k*_*i*_, *k*_*i*_ + 1, …, min(*n*_*i*_ − *l*_*i*_, *m*). The *concordance* for *x*_*i*_ is then given as:

$$\mathrm{con}c_{i}=\sum_{j=k_{i}}^{\min\left(n_{i}-1,m\right)}\left(\begin{array}{c} n_{i}-l_{i}\\ j \end{array}\right)p^{j}{\left(1-p\right)}^{n_{i}-j-l_{i}}$$

### Richness

The *richness* statistic for a HYP indicates the enrichment of nodes that have an observed significantly increased or decreased state in the downstream nodes of the network (*possible*) compared to the number of significantly increased or decreased RNAs (*state changes*) in the total *population* of measured RNAs mapped to HYPs. *Richness* is calculated as a p-value using a hypergeometric probability distribution, a method commonly used to characterize the degree to which a subset of a whole is particularly notable.

The hypergeometric distribution can be written as *f*(*k,N,m,n*) where:

*k* is the number of notable items in the subset (*observed*),

*N* is the number of items in the full set (*population*),

*m* is the number of notable items in the full set (*state changes*), and

*n* is the size of the subset (*possible*).

The probability that the subset is not due to chance can be calculated as:

$$\mathrm{prob}=f\left(k;N,m,n\right)=\frac{\left(\begin{array}{c} m\\ k \end{array}\right)\left(\begin{array}{c} N-m\\ n-k \end{array}\right)}{\left(\begin{array}{c} N\\ n \end{array}\right)}$$

For the *richness* calculation, the size of the full set, *N*, corresponds to the number of observations in the experiment that can be identified and mapped to at least one downstream node in the set of all possible HYPs (for a given HYP generation algorithm); *m* is the cardinality of the set of state changes. These values are constant for all mechanisms *x*_*i*_, *i* = 1,2,…,*n*. For any mechanism, *x*_*i*_, *n*_*i*_ is the number of downstream nodes that are mapped to measurements in the experiment (*possible*), and the number of notable items, *k*_*i*_, corresponds to the number of downstream nodes also found in the set of *m* state changes (*observed*).

The point probability for some HYP, *x*_*i*_, for some *k*_*i*_ is then calculated as:

$$\mathrm{pro}b_{\mathrm{ik}}=\frac{\left(\begin{array}{c} m\\ k_{i} \end{array}\right)\left(\begin{array}{c} N-m\\ n_{i}-k_{i} \end{array}\right)}{\left(\begin{array}{c} N\\ n_{i} \end{array}\right)}$$

The *richness* p-value is a cumulative probability based on the area under the curve of a probability distribution function. Thus, *richness* for a HYP, *x*_*i*_, is the sum of *prob*_*ij*_ for all *j* ≥ *k*_*i*_. Additionally, *j* is bounded by the size of the subset *n*_*i*_ and number of notable events *m*. The complete *richness* calculation is then given as:

$$\mathrm{ric}h_{i}=\sum_{j=k_{i}}^{\min\left(n_{i}\;m\right)}\frac{\left(\begin{array}{c} m\\ j \end{array}\right)\left(\begin{array}{c} N-m\\ n_{i}-j \end{array}\right)}{\left(\begin{array}{c} N\\ n_{i} \end{array}\right)}$$

which determines the cumulative probability over the range *j* = *k*_*i*_, *k*_*i*_ + 1, …, min(*n*_*i*_, *m*).

### Data analysis for examples

Three gene expression data sets were used as examples to evaluate Whistle output: (1) a mouse high fat diet insulin resistance model [24]; (2) human microvascular endothelial cells treated with TNF [25]; and (3) human breast epithelial cells treated with a PI3 kinase inhibitor [26]. Microarray data sets used as input for the TNF (GSE2638) and PIK3CA (GSE17785) examples were downloaded from Gene Expression Omnibus (GEO) (http://www.ncbi.nlm.nih.gov/gds). The high fat diet (E-MEXP-1755) data set was downloaded from ArrayExpress (http://www.ebi.ac.uk/arrayexpress/). GSE2638 used the Affymetrix Human Genome U133A, GSE17985 uses Affymetrix Human Genome U133 Plus 2.0, and E-MEXP-1755 used the Affymetrix Mouse genome 430 2.0 Array. Raw RNA expression data for each data set was analysed using the “affy” (2.10.0) and “limma” (2.10.1) packages of the Bioconductor suite of microarray analysis tools available for the R statistical environment [27-29]. Robust Microarray Analysis (RMA) background correction and quantile normalization were used to generate microarray expression values. An overall linear model was fit to the data for all sample groups, and specific contrasts of interest were evaluated to generate raw p-values for each probe set on the expression array [30].

Unless otherwise specified, state changes for probe sets were generated using the following significance criteria: (1) adjusted p-values of less than or equal to 0.05, corrected for multiple testing effects using Benjamini-Hochberg FDR, (2) absolute fold changes of at least 1.3, and (3) an average normalized Affymetrix abundance of at least 32. Other thresholds were used to determine differential probe sets as specified. Genes represented by multiple probe sets were considered to have changed if at least one probe set met criteria for differential expression. Gene expression changes that met these criteria are considered to be significantly differentially expressed and have the directional qualities of increased or decreased, i.e., they were upregulated or downregulated, respectively, in response to the experimental perturbation.

### Knowledge base

The “BEL Large Corpus”, version 1.4, (available at http://resource.belframework.org/belframework/1.0/knowledge/large_corpus.bel), was used as the prior knowledge source for the examples. The Large Corpus contains approximately 80,000 causal statements manually curated from the published biomedical literature, each statement representing an observation from experiments performed in human, mouse, or rat. Each statement is associated with a citation and key experimental context information (species, cell line, tissue, etc.). The Large Corpus was compiled into a Knowledge Assembly Model (KAM) using the BEL Framework, version 3.0.0 (available at http://download.openbel.org/OpenBEL_Framework-3.0.0-preview.zip) with the default compiler settings. For the examples, KAM nodes representing orthologous nodes were collapsed to either mouse or human as appropriate, using the BEL Framework API.

Because a BEL KAM is used as the prior knowledge source, each HYP is referred to by the BEL term label of its upstream node. BEL terms follow the general format *f(ns:v)*, where *f* is a function like ‘proteinAbundance’ (short form ‘p’) or ‘biologicalProcess’ (short form ‘bp’); *ns* is a reference to a namespace like ‘MGI’ (Mouse Genome Informatics symbol) or ‘EGID’ (Entrez Gene Id); and *v* is a value from the indicated namespace, like ‘Akt1’. A list of BEL functions and namespace abbreviations used in the examples is provided in Additional file 1. Full BEL language documentation can be found in the OpenBEL Portal (http://www.openbel.org/content/bel-lang-language).

## Results

### Example data sets

To highlight the utility of RCR for generating testable mechanistic hypotheses from gene expression profiling data, we provide examples of the application of Whistle v1.0 to three published gene expression data sets using a network compiled from the BEL Large Corpus as the prior knowledge source. Each data set was analysed to identify significantly differentially expressed probe sets (see *Methods*) and the resulting data analysed using Whistle and the Large Corpus KAM (Table 2). These examples demonstrate the use of Whistle to identify potential molecular upstream controllers of observed differential gene expression from experimental data sets.

**Table 2:** Overview of data sets used for examples

**Data Set**	**Reference PMID**	**Species**	**Platform**	**Perturbation**	**Significant probe sets**	**Population**	**State changes**	**Evaluated HYPs**	**Significant HYPs**
E-MEXP-1755	19196459	Mouse	MG-430 2.0	High fat diet	372	7594	193	606	13
GSE2638	16617158	Human	HG-U133A	TNF	557	6644	330	593	58
GSE17785	22570710	Human	HG-U133 Plus 2.0	GDC-0941 (PI3K inhibitor)	3410	7500	1126	603	45

Probe sets were considered significantly differential if average abundance > = 32, fold change > = 1.3, and adjusted p-value < = 0.05. The *population* size is defined as the number of unique measured RNA abundances mapped to HYPs. The number of *state changes* is defined as the number of differential RNAs in the *population*. Mechanisms were evaluated as HYPs if at least four measured RNAs are downstream nodes. HYPs were considered significant if both richness p < = 0.1 and concordance p < = 0.1.

### Example 1 – high fat diet diabetes model

We applied Whistle to data set E-MEXP-1755, the liver gene expression profile from mice fed a high fat diet, a model for impaired glucose tolerance and type 2 diabetes [31]. For this data set, C57BL/6 J male mice were fed either a high fat diet or standard chow for 15 weeks, and RNA was extracted from the liver for profiling [24].

Mapping of measurements from the Affymetrix MG-430 2.0 microarray supported the generation of 606 HYPs for evaluation from the mouse Large Corpus KAM (see Additional file 2), with a *population* of 7594 unique downstream RNA abundance nodes. The 372 probe sets meeting criteria for significant differential expression between high fat diet and normal diet mouse livers map to 193 unique RNA abundance nodes in the population (Table 2).

**Figure 3:**
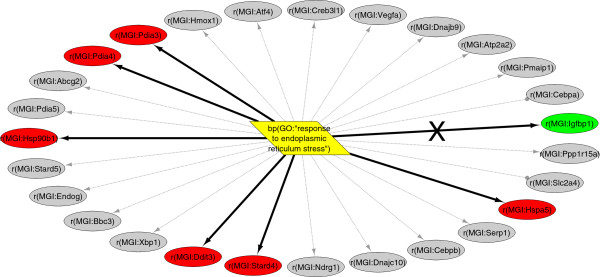
**Scored HYP example.** The HYP with the upstream node *bp(GO:“response to endoplasmic reticulum stress”)*, scored for the E-MEXP-1755 high fat diet data set. This network contains 27 measured RNA abundance nodes (*possible*), represented as ovals coloured by differential expression (red – significantly increased, green – significantly decreased, grey – no significant change). A total of seven differentially expressed RNAs mapped to the network (*observed*), including six supporting increased mechanism activity (*correct*) and one supporting decreased activity (*contra*, marked with an ‘X’ on the edge).

Of the 606 HYPs evaluated from the mouse Large Corpus KAM, 13 met the standard richness and concordance p-value significance thresholds of 0.1 (Table 3) (see *Randomized data sets* for threshold selection). For example, the HYP *bp(GO:“response to endoplasmic reticulum stress”)* is inferred to be significantly increased for the high fat data set (Figure 3). This mechanism, representing the biological process defined by the GO term ‘response to endoplasmic reticulum stress’, is causally upstream from 27 measured RNA abundances in the mouse-orthologized Large Corpus KAM (*possible*). Of the 193 significantly increased or decreased RNA abundance nodes resulting from high fat diet, seven map to the *bp(GO:“response to endoplasmic reticulum stress”)* HYP, representing a significant enrichment in endoplasmic reticulum stress-regulated RNA abundance nodes (*richness* p = 3.5E-6). Of these seven, six are in a direction consistent with increased response to endoplasmic reticulum stress, and one is consistent with decreased response. Thus, the *direction* increased is assigned to the *bp(GO:“response to endoplasmic reticulum stress”)* HYP. The *concordance* p-value, which evaluates the directions of the observed states of the downstream nodes against the predictions made by the HYP, is 6.3E-2, supporting the inference of increased *bp(GO:“response to endoplasmic reticulum stress”).*

**Table 3:** Significant HYPs for mouse high fat diet data set (E-MEXP-1755)

**Mechanism**	**Direction**	**Correct**	**Richness**	**Concordance**	**Ambiguous**	**Contra**	**Possible**	**Observed**
**a(SCHEM:“serum insulin”)**	**1**	**13**	**2.1E-04**	**1.1E-02**	**0**	**3**	**228**	**16**
**path(SDIS:“food intake”)**	**1**	**16**	**2.0E-07**	**2.6E-02**	**0**	**6**	**244**	**22**
**p(MGI:Ins2)**	**1**	**5**	**1.3E-03**	**3.1E-02**	**0**	**0**	**33**	**5**
**a(CHEBI:insulin)**	**1**	**5**	**3.6E-03**	**3.1E-02**	**0**	**0**	**41**	**5**
**kin(p(PFM:“Akt Family”))**	**1**	**5**	**8.4E-03**	**3.1E-02**	**0**	**0**	**50**	**5**
path(SDIS:“lung adenocarcinoma”)	1	8	4.0E-04	5.5E-02	0	2	108	10
tscript(complex(NCM:“AP-1 Complex”))	1	8	1.2E-02	5.5E-02	0	2	172	10
a(CHEBI:androgen)	1	11	8.4E-04	5.9E-02	0	4	233	15
bp(GO:“response to endoplasmic reticulum stress”)	1	6	3.5E-06	6.3E-02	0	1	27	7
bp(GO:“response to oxidative stress”)	−1	4	3.1E-02	6.3E-02	0	0	47	4
tscript(p(MGI:Foxa2))	−1	6	3.6E-02	6.3E-02	0	1	122	7
a(CHEBI:glucocorticoid)	−1	6	8.3E-02	6.3E-02	0	1	148	7
bp(GO:“response to starvation”)	−1	7	4.4E-04	9.0E-02	0	2	90	9

Thirteen HYPs meet both richness and concordance thresholds of p < = 0.1 for data set E-MEXP-1755. Five mechanisms meet more stringent p < 0.05 thresholds (in bold).

Several of the significant mechanisms inferred by Whistle are consistent with the dietary changes and insulin resistance associated with this mouse model. Increased food intake (*path(SDIS:“food intake”)*) and decreased response to starvation (*bp(GO:“response to starvation”)*) are consistent with the dietary differences characterizing the high-fat diet group and the control group. Circulating insulin levels are reported to be increased in the high fat diet fed mouse model [31], and several HYPs representing increased insulin (*p(MGI:Ins2), a(CHEBI:insulin),* and *a(SCHEM:“serum insulin”)*) are inferred by Whistle. Increased response to endoplasmic reticulum stress (*bp(GO:“response to endoplasmic reticulum stress”)* is consistent with reports of ER stress induction in mouse obesity models [32]. The decreased transcriptional activity of Foxa2 protein (*tscript(p(MGI:Foxa2))*) is consistent with the reported inactivation of Foxa2 in insulin-resistant mouse models [33].

Several of the highly relevant HYPs inferred by Whistle for the E-MEXP-1755 data set represent the abundance or activity of proteins for which the corresponding RNAs are not differentially expressed. These mechanisms would not be identified by standard overrepresentation analyses based on the functions of the proteins encoded by the differentially expressed genes. For example, insulin (represented by the HYPs *p(MGI:Ins2), a(CHEBI:insulin),* and *a(SCHEM:“serum insulin”)*) is inferred increased, but probe sets for the mouse insulin genes Ins1 and Ins2, and the insulin receptor gene Insr do not meet criteria for differential expression. Insulin is produced by the pancreas, and is not expected to be regulated by gene expression in the liver. Similarly, the HYP representing the transcriptional activity of Foxa2, *tscript(p(MGI:Foxa2))*, is inferred decreased, but the Foxa2 RNA (probe set 1422833_at) is not differentially expressed. Foxa2 is an insulin-regulated liver transcription factor whose activation leads to oxidation of fatty acids and secretion of triacyglycerols, and whose impairment has been implicated in diabetes [34]. Foxa2 regulation by insulin occurs via phosphorylation [33], consistent with the lack of observed regulation of Foxa2 RNA.

### Example 2 – TNF treatment of human microvascular endothelial cells

We applied Whistle to a gene expression profile of the response of human microvascular endothelial cells to TNF. This data set (GSE2638) was generated by stimulating HMEC-1 cells with 2 ng/ml TNF for 5 hours [25]). In endothelial cells, TNF, a potent mediator of inflammatory signalling, acts through its cognate receptors to initiate downstream signalling, including activation of the transcription factor NF-κB, resulting in increased transcription of cell surface receptors involved in leukocyte adhesion [35-37].

Mapping of measurements from the Affymetrix U133A microarray supported the generation of 593 HYPs from the human Large Corpus KAM (see Additional file 2), with a population of to 6644 unique downstream RNA abundance nodes. The 557 probe sets meeting criteria for significant differential expression between TNF stimulated and untreated cells map to 330 unique RNA abundance nodes in the *population*.

Of the 593 HYPs evaluated from the human Large Corpus KAM, 58 met the standard *richness* and *concordance* p-value significance thresholds of 0.1. The top 20 significant mechanisms as ranked by the *concordance* statistic are shown in Table 4. These significant mechanisms include several molecules and processes canonically associated with TNF signalling. Transcriptional activity of NF-κB complex (*tscript(complex(PFH:“Nfkb Complex”))*) is inferred increased, consistent with the complex’s role as a key mediator of TNF-induced transcription [36]. TNF reduces endothelial cell lifespan [38], consistent with the inferred increase of replicative cell aging (*bp(GO:“replicative cell aging”)*). TNF activates p38 MAPK in endothelial cells [37], and increased kinase activity of p38 MAPK (*kin(p(PFH:“p38 MAPK Family”))*) is inferred by Whistle. Interestingly, while the inferred increases of lipopolysaccharide (*a(CHEBI:lipopolysaccharide)*) bacterial infection (*path(SDIS:“bacterial infection”)*) seem biologically implausible to occur during the 5 hour TNF treatment used to generate the data set, these mechanisms are aligned with reports that LPS and TNF activate very similar transcriptional responses in endothelial cells [39]. A HYP representing TNF itself was not evaluated, as TNF is not connected to sufficient downstream RNA abundance nodes in the Large Corpus KAM.

**Table 4:** Top 20 significant HYPs for TNF data set (GSE2638)

**Mechanism**	**Direction**	**Correct**	**Richness**	**Concordance**	**Ambiguous**	**Contra**	**Possible**	**Observed**
tscript(complex(NCH:“Nfkb Complex”))	1	69	6.2E-25	9.2E-16	2	5	417	76
path(SDIS:“bacterial infection”)	1	34	1.6E-10	1.0E-09	0	1	211	35
bp(GO:“replicative cell aging”)	1	33	3.1E-19	1.1E-07	0	3	121	36
p(PFH:“IFNA Family”)	1	25	4.0E-12	4.0E-07	0	1	105	26
a(CHEBI:lipopolysaccharide)	1	26	1.8E-06	7.6E-06	0	3	225	29
tscript(complex(NCH:“AP-1 Complex”))	1	19	2.1E-04	2.0E-05	0	1	167	20
path(SDIS:“tissue damage”)	1	19	1.2E-03	2.0E-05	0	1	191	20
a(SCHEM:“Dietary Lipid”)	1	14	1.0E-07	6.1E-05	0	0	51	14
a(SCHEM:Allergens)	1	17	4.5E-08	7.2E-05	0	1	80	18
bp(GO:angiogenesis)	1	20	6.7E-09	2.4E-04	0	3	115	23
p(HGNC:AGT)	1	17	4.0E-06	3.6E-04	0	2	117	19
tscript(p(HGNC:TP63))	1	17	1.2E-05	3.6E-04	0	2	126	19
kin(p(PFH:“PRKC Family”))	1	17	4.2E-04	3.6E-04	0	2	163	19
kin(p(PFH:“MAPK p38 Family”))	1	14	6.8E-09	4.9E-04	0	1	49	15
a(SCHEM:“Tetradecanoylphorbol acetate”)	1	16	2.5E-04	6.6E-04	0	2	144	18
bp(GO:“response to hypoxia”)	1	49	7.8E-05	9.1E-04	1	22	943	72
path(SDIS:“Photo-oxidative Stress”)	1	10	3.6E-06	9.8E-04	0	0	34	10
a(CHEBI:“arachidonic acid”)	1	15	2.6E-07	1.2E-03	0	2	80	17
tscript(p(HGNC:MEOX2))	−1	17	8.6E-10	1.3E-03	0	3	79	20
a(SCHEM:“Oxidized Low Density Lipoprotein”)	1	17	1.3E-04	1.3E-03	0	3	161	20

Fifty-eight HYPs met richness and concordance thresholds of p < = 0.1 for data set GSE2638; the top twenty significant HYPs, as ranked by concordance are shown.

### Example 3 – PI3K inhibitor treatment of human breast epithelial cells

We applied Whistle to data set GSE17785, the gene expression profile of human breast epithelial cells treated with a PI3K inhibitor. For this data set, a clone of cell line MCF10A with a knock-in of the activating PIK3CA mutation H1047R was treated with either the PI3K inhibitor GDC-0941 or the vehicle DMSO for 4 hours [26]. The phosphatidylinositol 3-kinase (PI3K) signalling pathway is a key mediator of cell survival and proliferation [40].

Mapping of measurements from the Affymetrix U133 Plus 2.0 microarray supported the generation of 603 HYPs from the human Large Corpus KAM (see Additional file 1), with a *population* of 7500 unique downstream RNA abundance nodes. The 3410 probe sets meeting criteria for significant differential expression between PI3K inhibitor-treated and DMSO-treated cells map to 1126 unique RNA abundance nodes in the *population*.

Of the 603 mechanisms evaluated from the human Large Corpus KAM, 45 met the standard *richness* and *concordance* p-value significance thresholds of 0.1, including decreased PI3K complex activity (*kin(complex(NCH:“p85/p100 PI3Kinase Complex”))*) and several mechanisms canonically associated with inhibition of PI3K signalling, e.g., increased activity of FOXO transcription factors FOXO1, FOXO3, and FOXO4 (*tscript(p(HGNC:FOXO1))*, *tscript(p(HGNC:FOXO3*)), *tscript(p(HGNC:FOXO4))*); decreased AKT kinase activity (*kin(p(PFH:“AKT Family”))*), and decreased insulin signalling (*a(SCHEM:“serum insulin”)*) [40]. The top 20 significant mechanisms as ranked by the *concordance* statistic are shown in Table 5.

**Table 5:** Top 20 significant HYPs for the PI3K inhibitor data set (GSE17785)

**Mechanism**	**Direction**	**Correct**	**Richness**	**Concordance**	**Ambiguous**	**Contra**	**Possible**	**Observed**
a(CHEBI:lapatinib)	1	21	1.5E-03	4.8E-07	0	0	72	21
tscript(p(HGNC:FOXO1))	1	43	2.2E-05	7.0E-06	0	11	208	54
tscript(p(HGNC:FOXO3))	1	34	1.6E-10	3.4E-05	0	8	103	42
a(SCHEM:Hydrocortisone)	1	41	2.4E-07	4.1E-05	0	12	177	53
a(CHEBI:estradiol)	−1	36	1.4E-03	7.8E-05	0	10	199	46
a(SCHEM:“serum insulin”)	−1	46	1.5E-07	8.8E-05	0	16	217	62
kin(p(PFH:“PRKC Family”))	−1	26	3.3E-02	1.5E-03	0	8	166	34
a(CHEBI:“nitric oxide”)	−1	29	8.2E-03	1.7E-03	0	10	178	39
path(SDIS:“lung adenocarcinoma”)	−1	20	1.3E-02	2.0E-03	0	5	106	25
bp(MESHPP:“Menstrual Cycle”)	1	18	5.2E-03	2.2E-03	0	4	84	22
a(CHEBI:camptothecin)	−1	23	3.1E-04	5.3E-03	0	8	111	31
p(HGNC:AGT)	−1	23	1.1E-03	5.3E-03	0	8	119	31
tscript(p(HGNC:FOXO4))	1	7	5.6E-03	7.8E-03	0	0	16	7
tscript(p(HGNC:SP5))	1	17	3.2E-03	8.5E-03	0	5	81	22
a(CHEBI:androgen)	−1	35	8.0E-04	8.8E-03	0	17	226	52
bp(GO:“response to heat”)	−1	39	1.2E-05	9.2E-03	0	20	229	59
path(SDIS:“viral infection”)	1	42	3.3E-08	1.2E-02	0	23	223	65
kin(complex(NCH:“p85/p110 PI3Kinase Complex”))	−1	22	2.5E-06	1.5E-02	0	9	89	31
kin(p(PFH:“AKT Family”))	−1	14	7.7E-05	1.5E-02	1	4	51	19
a(CHEBI:haloperidol)	−1	6	2.4E-02	1.6E-02	0	0	16	6

Forty-five HYPs met both richness and concordance p < = 0.1 thresholds for data set GSE17785. The top twenty HYPs, as ranked by concordance are shown.

### Randomized data sets

Random data are not expected to produce a biological signal. To further investigate the significance of the mechanisms inferred by Whistle, we generated a set of 1,000 matched random data sets for each example data set and compared the Whistle results to those for the matched real data. These random data sets were generated such that the *population* was the same as the matched example data set and that the total number of significantly increased and decreased RNAs in the randomized data matched that of the real data set.

**Figure 4:**
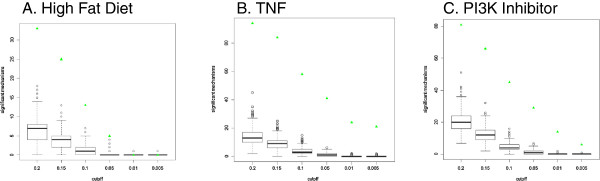
**Numbers of significant HYPs at different richness and concordance thresholds in randomized data sets.** Boxplot showing the number of HYPs (mechanisms) meeting the specified richness and concordance threshold across 1,000 randomized data sets. The number of significant HYPs at each threshold for the matching real data is plotted for comparison in green. **(A)** High Fat Diet, **(B)** TNF treatment, **(C)** PI3K inhibitor.

We used the random data Whistle results to guide the selection of a generally applicable threshold for *richness* and *concordance* p-values to use to identify significant HYPs, minimizing false positive and false negative significant HYPs. Whistle results for the randomized data sets supported the selection of 0.1 as a standard threshold for *richness* and *concordance* p-values. Applying thresholds of p < = 0.1 for both *richness* and *concordance* to all three example data sets and their matched random data yields a median number of significant HYPs inferred for the 1,000 random data sets equal to 5-10% of the number of HYPs inferred for the corresponding real data at the same threshold (Table 6, Figure 4). Thus, we expect this threshold to yield less than 10% false positive inferred HYPs for most data sets. While selection of a more stringent p-value threshold such as 0.05 reduced the number of HYPs meeting significance criteria in the random data sets, it resulted in false negatives, i.e., the loss of several biologically relevant mechanisms. Specifically, lowering the p-value threshold from 0.1 to 0.05 for the high fat diet data set resulted in the loss of eight of the thirteen HYPs including increased *bp(GO:“response to endoplasmic reticulum stress”)*, decreased *bp(GO:“response to starvation”)*, and decreased *tscript(p(MGI:Foxa2))*. As the mechanisms represented by these HYPs are highly biologically relevant for the high fat data set (see *Example 1*), we chose to apply the relatively permissive p < = 0.1 *richness* and *concordance* evaluation statistic threshold.

**Table 6:** HYPs meeting significance criteria in matched real and randomized data sets

**Richness and concordance p-value threshold**	**TNF**			**PI3K inhibitor**			**High fat diet**		
	**Random**	**Real**	**Ratio**	**Random**	**Real**	**Ratio**	**Random**	**Real**	**Ratio**
0.2	13	94	14%	20	81	25%	7	33	21%
0.15	9	84	11%	12	66	18%	4	25	16%
**0.1**	**3**	**58**	**5%**	**4**	**45**	**9%**	**1**	**13**	**8%**
0.05	1	41	2%	1	29	3%	0	5	0%
0.01	0	24	0%	0	14	0%	0	0	N/A
0.005	0	21	0%	0	6	0%	0	0	N/A

For each example data set (TNF, PI3K inhibitor, and high fat diet), 1000 matched random data sets were generated. The number of HYPs meeting significance criteria at each p-value threshold is indicated, for random data the median number of significant mechanisms is shown.

BOLD indicates the 0.1 p-value threshold applied to analysis of the three example data sets.

### Bias in evaluation statistics by HYP size

We further evaluated the *richness* and *concordance* statistics by examining bias of both metrics to the number of downstream RNAs (*possible*) in the HYP within the randomized data sets. Because these data sets do not contain true biological signal, the correlation of either metric with the HYP size can be used to examine the expected bias.

### Richness

Minimal correlation between *richness* and HYP size (*possible*) was observed in the randomized data (Pearson correlation coefficient −0.125; see Additional file 3). This weak degree of correlation suggests that the HYP size has minimal influence on *richness*.

### Concordance

A higher level of correlation between concordance and HYP size was observed in the randomized data (Pearson correlation coefficient −0.457; see Additional file 3: Figure S5).

To correct this bias, we fit a LOESS curve to the log concordances from a subset of the randomized data matching the example data sets (n = 180,300 HYP scores) and computed an adjusted concordance measure by subtracting the fit from the log raw concordance. This correction reduced the overall correlation of concordance to the HYP size (*possible*) in each of the three example data sets (see Additional file 3: Figure S7). The Spearman correlation of the top 50 HYPs by concordance versus adjusted concordance was 0.80, 0.97, and 0.92 for the High Fat Diet (E-MEXP-1755), TNF (GSE2638), and PI3K Inhibitor (GSE17785) data sets, respectively. The corrected concordance values had minimal effect on the ranking of the top HYPs by concordance in the three example data sets.

Moreover, limiting the HYPs to those which are associated with a minimum number of differentially expressed RNAs removes much of the concordance bias in the randomized data. Concordance evaluates the consistency of the direction of the significant RNA expression changes with the HYP sub-network and thus is most relevant when applied to HYPs which have several significantly changing RNAs. The *richness* statistic is used to evaluate if the number of differentially expressed RNAs for a given mechanism is significant. In practice, concordance is critically evaluated only for those mechanisms for which at least four downstream RNAs are differentially expressed (*observed*), as this number is the minimum required for concordance to meet the 0.1 threshold. In the randomized data sets, only about 20% of total scored HYPs have at least four *observed*. Limiting to these scores reduces the correlation coefficient between *concordance* and *possible* in the randomized data from −0.457 to −0.181 (see Additional file 3: Figure S6). While the concordance bias is significant, it has limited impact on the use of concordance to filter the HYPs to a list of interesting mechanisms for a data set or to use concordance to rank the most interesting HYPs. Given the limited influence of the concordance bias within the range of interest, we have not implemented correction of concordance in Whistle.

## Discussion

RCR is a reverse engineering algorithm that reasons from observed effects to potential causes, transforming lists of differentially expressed genes into mechanistic hypotheses. The use of a knowledge base structured as a directed, causal network provides RCR with some key advantages over other analysis techniques relying on prior knowledge: (1) RCR does not rely on the assumption that changes in RNA expression are equivalent to changes in the activity of the corresponding protein, (2) the structuring of gene sets as networks (‘HYPs’) allows evaluation of genes up- and down-regulated by the same mechanism as a cohesive, causally consistent mechanism, and (3) flexibility to generate HYP networks for evaluation from the knowledge base network, potentially combining related upstream nodes to a single HYP or dividing HYPs based on knowledge context.

The integration of qualitative causal relationships in Reverse Causal Reasoning fundamentally distinguishes it from other techniques in which gene expression profiling data is interpreted via over-representation analysis of functionally related sets of genes. The HYP networks assessed by RCR group measurable quantities based on a shared upstream controller, and specify the direction of control for each measurable quantity. Gene sets for over-representation analysis derived from pathway maps such as KEGG [4] or gene ontology (GO) annotation [5], differ sharply from the HYPs used by RCR in that the genes within a gene set are not related by a specified common regulator. Gene sets derived from experimental data (e.g., L2L, [9]) are more similar to mechanism networks because they are selected by a common cause (the perturbation in the experiment) but they generally do not express the causal information in a single structure. The incorporation of this causal information allows RCR to assess genes both up- and down-regulated by a controller as a common mechanism, and evaluate the causal consistency of the network against the observed data using the concordance metric.

RCR provides a qualitative assessment of significance of a mechanism, ideal for exploratory analyses to provide qualitative hypotheses about molecular mechanisms connecting the perturbation (e.g., disease or drug treatment) to the observed gene expression. In contrast, Network Perturbation Amplitude (NPA) methods, which use the same network structure as RCR to evaluate mechanisms, provide quantitative comparison of the activity of interesting mechanisms between data sets [21]. The RCR scores and evaluation metrics do not indicate relative strength of activation or inhibition of a mechanism, only the direction and significance. HYPs with lower richness and concordance p-values are more likely to be biologically relevant. We found that filtering to p-values below 0.1 typically results in a reasonable set of mechanisms for further manual evaluation, with an acceptably low level of false positives. Of note, HYPs with a relatively small number of downstream RNAs (possible) are intrinsically biased towards less significant p-values than larger HYPs due to the smaller sample size.

RCR shares a limitation with other analysis approaches that require prior knowledge that only those mechanisms or pathways that are represented in the body of prior knowledge can be inferred to be active. For example, the Whistle analysis of the TNF data set (Example 2) did not infer TNF itself to be significant. TNF is not connected to sufficient downstream RNA abundance nodes in the Large Corpus KAM, thus no HYP representing TNF was evaluated. The knowledge base used for the example data sets, the Large Corpus KAM, is significantly smaller than the knowledge base that Selventa routinely uses with this algorithm. The species-orthologized Large Corpus KAM generates approximately 600 mechanism networks. In contrast, the full Selventa knowledge base permits evaluation of more than 2000 mechanisms, and contains more downstream elements for each mechanism. We selected the Large Corpus KAM to use here because it is publically available and sufficiently large for demonstration purposes.

The derivation of HYP networks from a larger causal network provides two immediate logical extensions to RCR. First, individual HYP networks can be causally connected within the larger network (KAM). Each HYP represents a node in the KAM, thus HYPs that meet significance criteria can be joined through causal relationships in the KAM to generate more complex explanatory networks. For example, for the PI3K inhibitor treatment data set, decreased PI3K activity (*kin(complex(NCH:“p85/p110 PI3Kinase Complex”))*), decreased AKT kinase activity (*kin(p(PFH:“AKT Family”))*), and increased activity of FOXO transcription factors FOXO1, FOXO3, and FOXO4 (*tscript(p(HGNC:FOXO1))*, *tscript(p(HGNC:FOXO3*)), *tscript(p(HGNC:FOXO4))*) can be causally joined. Second, while Whistle is designed to create and score very simple HYP networks from a larger network, HYPs representing more complex networks can be derived from the KAM and evaluated by RCR. For example, algorithms could be created to generate HYPS representing transcriptional motifs like sets of genes co-regulated by multiple transcription factors, or signalling pathway motifs like a ligand and receptor and their combined set of downstream RNA targets.

## Conclusions

RCR, a reverse engineering algorithm, provides mechanistic explanations for molecular profiling data sets, reducing data complexity and providing hypotheses to guide follow-up experimentation. This approach of identifying upstream causes that can explain the data offers some advantages over methods that map differential entities to gene ontology categories or pathway maps, because it does not rely on the assumption that changes in RNA expression leads to comparable changes in active protein.

To identify potential explanations for a set of differential measurements obtained by comparison of two sample groups, this algorithm evaluates small networks representing biological mechanisms. These small networks are comparable to gene sets, but differ in their network structure that incorporates the direction of causal influence (increase or decrease) of the mechanism on the measured quantity. This network structure enables both evaluations incorporating the direction of influence and the possibility to link multiple significant networks into a larger explanatory network.

Whistle, an implementation of RCR focused on the interpretation of gene expression data that uses prior knowledge in BEL format, is available at (https://github.com/Selventa/whistle).

## Competing interests

All authors are either current or former employees of Selventa (formerly Genstruct). Selventa has applied for a patent related to the method described in this manuscript; TS and DP are listed as inventors on the application.

## Authors’ contributions

TS, WL, KOE, and DP developed the RCR methodology and initial implementation. AJB and SU developed Whistle. NLC provided requirements for and testing of Whistle and developed the Whistle/RCR use examples. NLC and DP drafted the manuscript. All authors read and approved the final manuscript.

## Supplementary Material

Additional file 1 BEL term and namespace abbreviations. Click here for file

Additional file 2 Evaluated HYPs for the TNF (HG- U133A), PI3K inhibitor (HG- U133 Plus 2.0), and high fat diet (MG-430 2.0 Array) data sets. Click here for file

Additional file 3: Figure S5 Evaluation of richness correlation with HYP size. Scatter plot (left) and boxplot (right) of *richness* versus HYP size (*possible*) for randomized data matched to the example data sets. Pearson correlation coefficient = -0.125. **S6.** Evaluation of concordance correlation with HYP size. Scatter plot (top left) and boxplot (top right) of *concordance* versus HYP size (*possible*) for randomized data matched to the example data sets. Pearson correlation coefficient = -0.457. Boxplot (bottom right) shows reduced correlation for scores limited to those HYPs with at least four RNA expression changes; correlation coefficient -0.181. **S7.** Evaluation of LOESS fit-adjusted concordance for example data sets.Boxplots for *concordance* versus HYP size (*possible*) for the High Fat Diet, TNF, and PI3K inhibitor example data sets (left) and adjusted concordance (right). Click here for file
