# Supplementary figures and images for: Reverse causal reasoning: applying qualitative causal knowledge to the interpretation of high-throughput data
Source: BMC Bioinformatics. 2013 Nov 23;14:340. doi: 10.1186/1471-2105-14-340 (PMC4222496; doi:10.1186/1471-2105-14-340)

Supplemental Figures

Figure 5

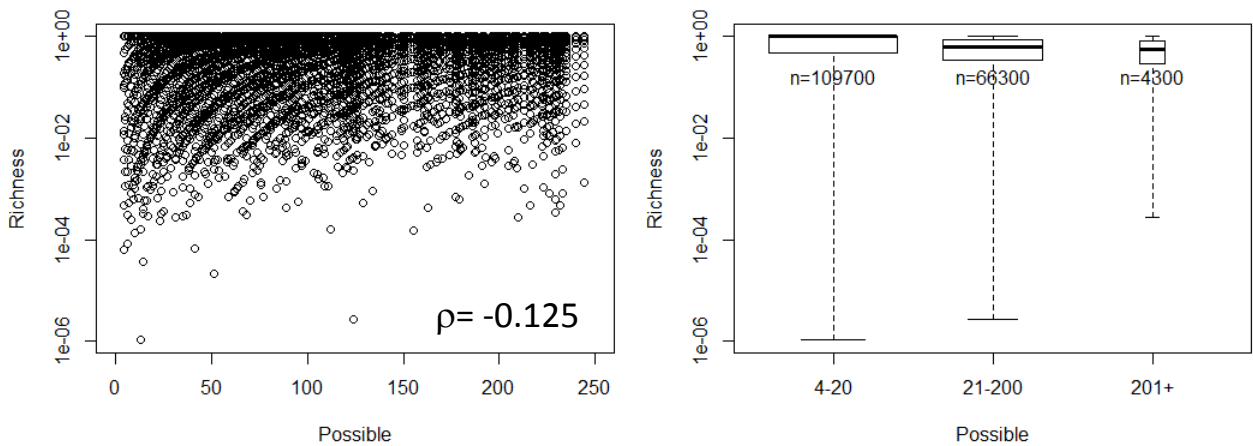

Figure 6

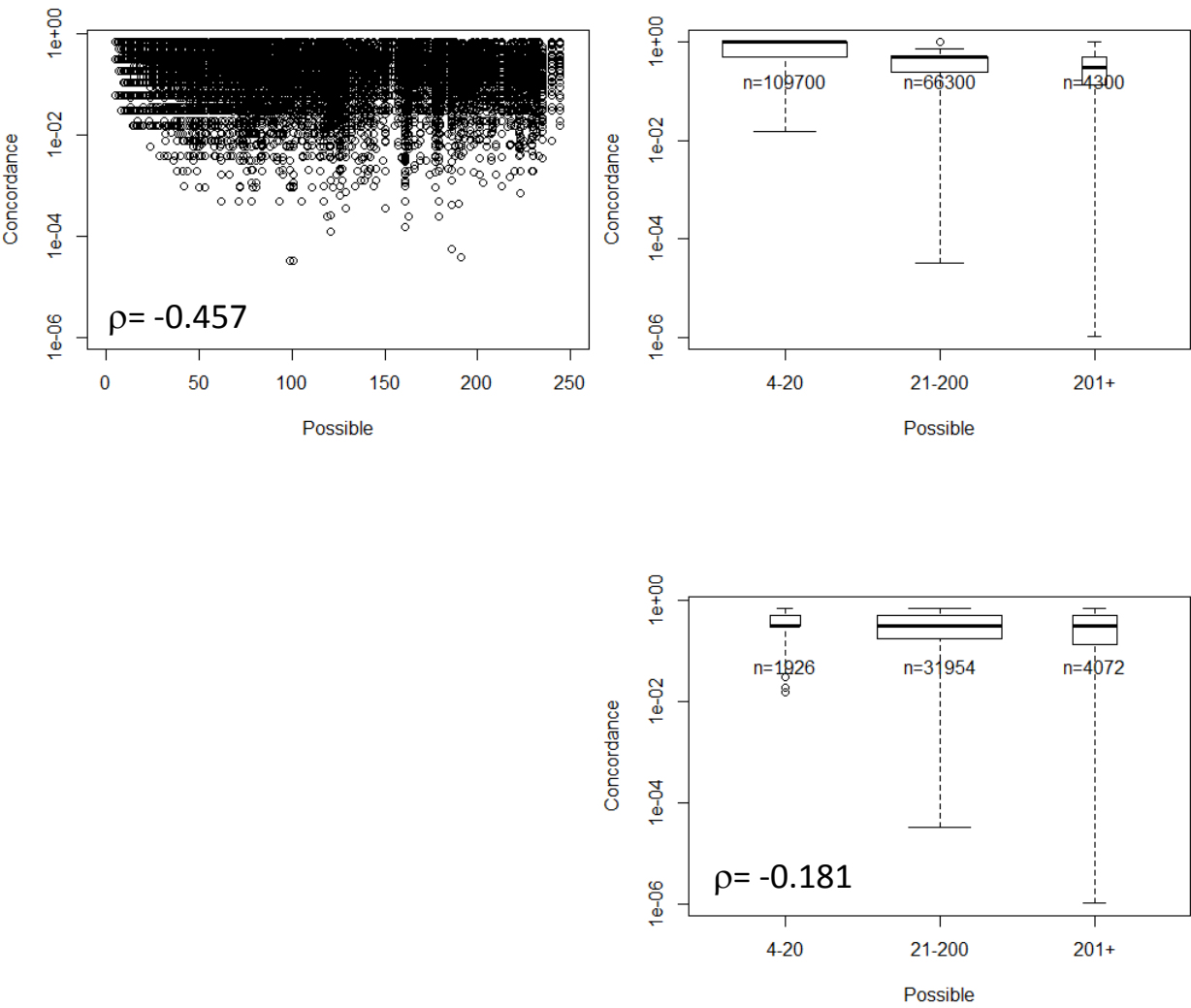

Figure 7

High Fat Diet (E-MEXP-1755)

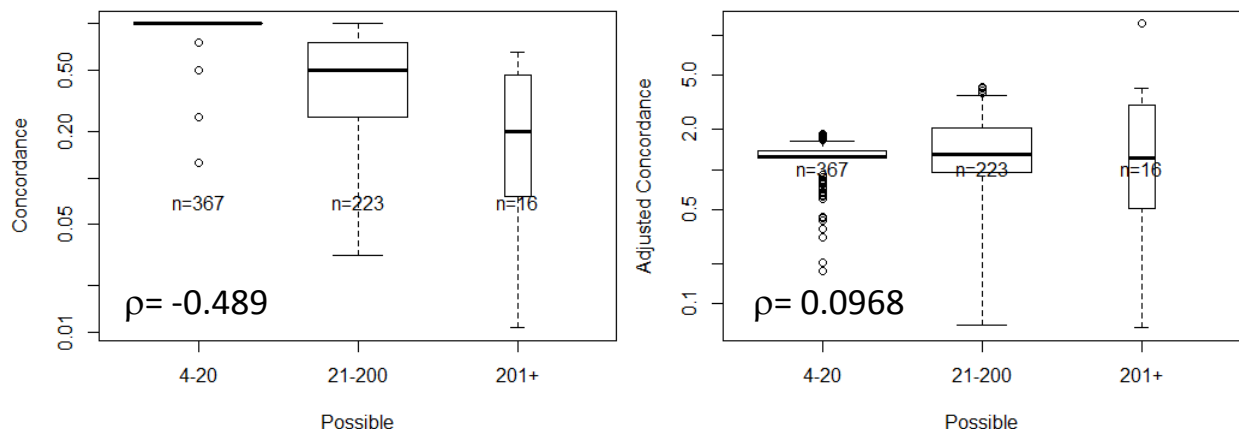

TNF (GSE2638)

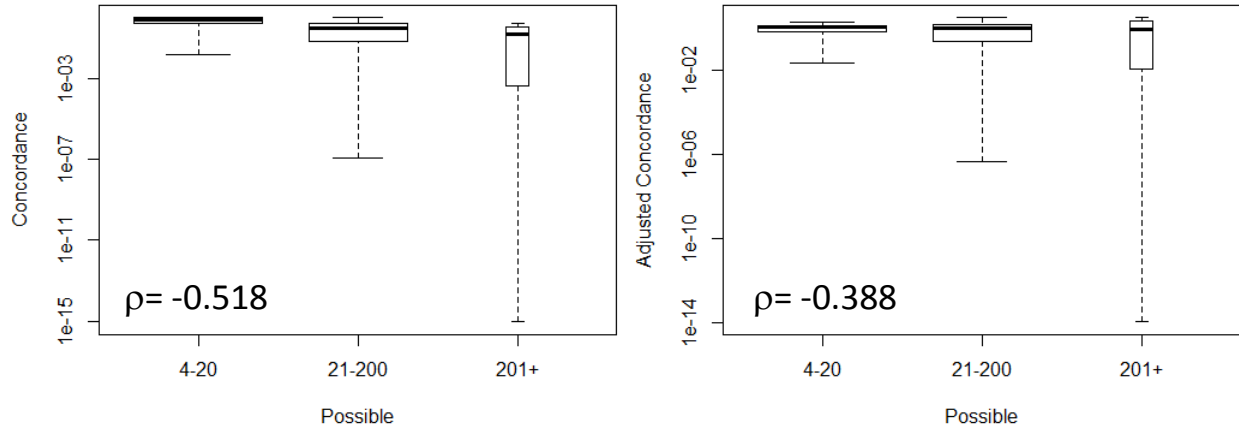

PI3K Inhibitor (GSE17785)

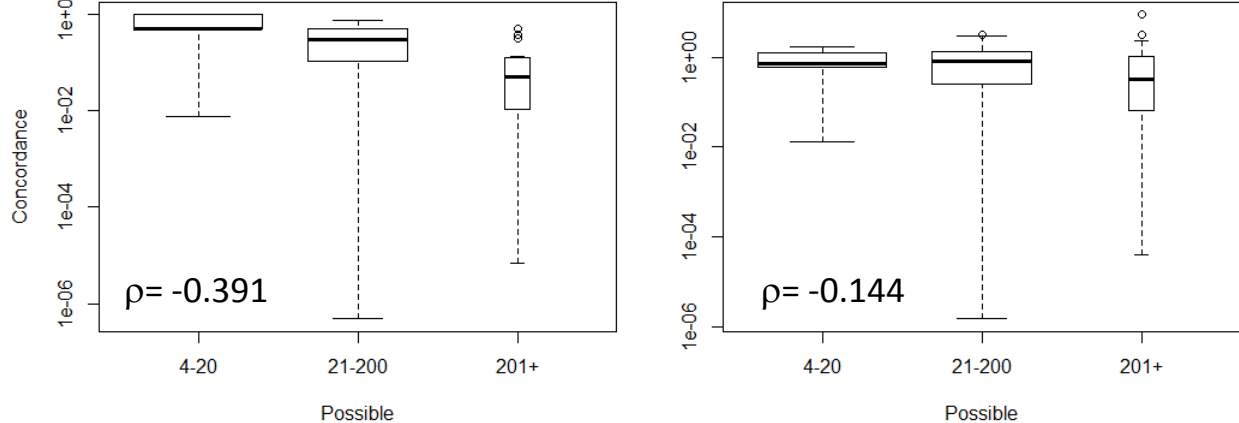

Supplement: Additional file 3: Figure S5 — Evaluation of richness correlation with HYP size. Scatter plot (left) and boxplot (right) of richness versus HYP size (possible) for randomized data matched to the example data sets. Pearson correlation coefficient = -0.125. S6. Evaluation of concordance correlation with HYP size. Scatter plot (top left) and boxplot (top right) of concordance versus HYP size (possible) for randomized data matched to the example data sets. Pearson correlation coefficient = -0.457. Boxplot (bottom right) shows reduced correlation for scores limited to those HYPs with at least four RNA expression changes; correlation coefficient -0.181. S7. Evaluation of LOESS fit-adjusted concordance for example data sets.Boxplots for concordance versus HYP size (possible) for the High Fat Diet, TNF, and PI3K inhibitor example data sets (left) and adjusted concordance (right). [file 1471-2105-14-340-S3.pdf]
